# Computer-aided analysis of radiological images for cancer diagnosis: performance analysis on benchmark datasets, challenges, and directions

**DOI:** 10.1186/s41824-024-00195-8

**Published:** 2024-04-01

**Authors:** Jaber Alyami

**Affiliations:** 1 Department of Radiological Sciences, Faculty of Applied Medical Sciences, King Abdulaziz University, 21589 Jeddah, Saudi Arabia; 2 King Fahd Medical Research Center, King Abdulaziz University, 21589 Jeddah, Saudi Arabia; 3 Smart Medical Imaging Research Group, King Abdulaziz University, 21589 Jeddah, Saudi Arabia; 4 Medical Imaging and Artificial Intelligence Research Unit, Center of Modern Mathematical Sciences and its Applications, King Abdulaziz University, 21589 Jeddah, Saudi Arabia

**Keywords:** Radiological images, MRI, Analysis, Clinical research applications, Cancer diagnosis, Multi-organs, Biopsy

## Abstract

Radiological image analysis using machine learning has been extensively applied to enhance biopsy diagnosis accuracy and assist radiologists with precise cures. With improvements in the medical industry and its technology, computer-aided diagnosis (CAD) systems have been essential in detecting early cancer signs in patients that could not be observed physically, exclusive of introducing errors. CAD is a detection system that combines artificially intelligent techniques with image processing applications thru computer vision. Several manual procedures are reported in state of the art for cancer diagnosis. Still, they are costly, time-consuming and diagnose cancer in late stages such as CT scans, radiography, and MRI scan. In this research, numerous state-of-the-art approaches on multi-organs detection using clinical practices are evaluated, such as cancer, neurological, psychiatric, cardiovascular and abdominal imaging. Additionally, numerous sound approaches are clustered together and their results are assessed and compared on benchmark datasets. Standard metrics such as accuracy, sensitivity, specificity and false-positive rate are employed to check the validity of the current models reported in the literature. Finally, existing issues are highlighted and possible directions for future work are also suggested.

## Introduction

Radiological image analysis is critical in detecting abnormalities in several body organs, including breast and lung cancer. Cancer is a disease that wreaks havoc on the cells in human bodies. Several cell types in the body contribute to the development of various forms of cancer. Cancer is caused by abnormal cell development, which spreads quickly. A collection of cancer cells will develop a tumour that will invade healthy tissues (Alyami et al. 2022a). Therefore, early identification is the most promising method of increasing a patient's persistence chances. Tumour cells are classed as benign or malignant according to their characteristics. Malignant tumours are cancerous, but benign tumours do not damage the surrounding cells.

Artificial intelligence (AI) technologies could change the healthcare field as shown by the extraordinary success of machine vision techniques, particularly deep learning applications in medical image analysis and diagnosis (Mughal et al. 2018; Saba et al. 2018a). This success may be attributed to the release of large and curated imaging corpora (probably the best-known of which is ImageNet), resulting in more oncology research with applications in tumour identification, genetic characterization and tumour subtyping, grading diagnosis, healthcare and health risk (Alyami and Nassef 2022). The possible applications span the whole medical imaging life cycle, from image acquisition to diagnosis and prediction of cancer (Yousaf et al. 2019). A recent survey of the literature on artificial intelligence in healthcare highlighted five areas in which AI is predicted to impact significantly: healthcare system management, diagnostics, clinical decision-making, teleradiology and predictive medicine (Sayad et al. 2021; Larabi-Marie-Sainte et al. 2019).

**Fig. 1:**
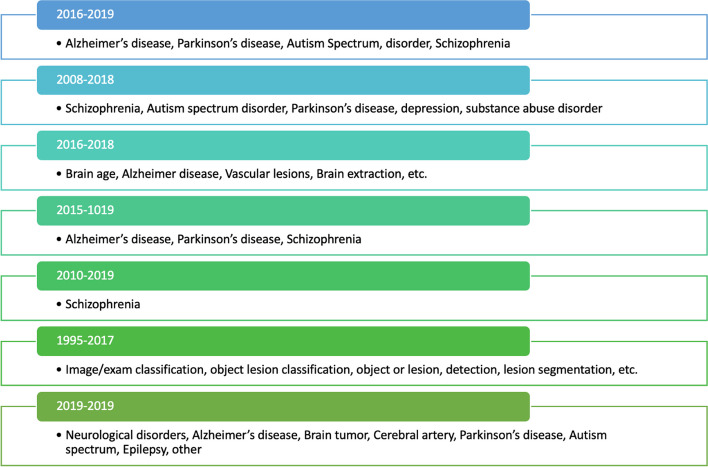
Application of CAD systems in healthcare

In the presence of sufficient input data, machine learning algorithms are capable of rapidly producing correct generalizations (Rehman et al. 2022; Rehman and Saba 2014). Random forests, clustering, and deep learning are just a few of the many machine learning techniques that have emerged since the 1990s. An emerging field, deep learning (Javed and Rahim 2020; Muhsin et al. 2014) allows computers to generalize input datasets without resorting to a predetermined set of rules. As an alternative, features are used to train classifiers to learn shared characteristics across datasets. The evolution of the healthcare industry's use of CAD systems is depicted in Fig. 1.

Cancer, which can metastasize (spread to other parts of the body) uncontrollably and ultimately prove fatal, is one of the most frequently diagnosed disorders worldwide. Detecting cancer at its earliest stages allows for more time to administer life-saving treatments and a lower risk of the disease progressing. Histopathology images confirm the stage of cancer with precise information and can be used to diagnose the disease from highly complex visual tissue. The following major gaps are identified based on the reviewed literature that need to be addressed by the research community.

First, so far, reported techniques on cancer diagnosis used hybrid segmentation methods using traditional machine learning. However, deep learning has shown state-of-the-art results in classification problems. Therefore, there is an emergent need to apply and explore deep learning models on medical images for cancer diagnosis at an early stage.

Secondly, the clinical applications cannot diagnose cancer at its early stages and this area of research is still fresh for research.

Based on the gap discussed earlier, the main contributions of this research are

1. Explored commonly employed imaging modality in multi-organs cancer diagnosis.
2. Investigated different databases in medical image analysis for cancer diagnosis.
3. Compared machine learning and deep learning classifiers' efficiency on benchmark datasets using different performance evaluation metrics.

The further paper is organized into four main sections. Section "AI-based systems for cancer diagnosis" highlights the worth of AI-based medical images analysis and classification for cancer diagnosis; "Limitations, pitfalls and future challenges" section mentions limitations, pitfalls and future challenges. Section "Open access benchmark datasets" details benchmark datasets available for experiments and finally, "Conclusion" section concludes the research.

## AI-based systems for cancer diagnosis

### Cancer imaging

Despite significant developments in cancer diagnosis, its treatment has been one of humanity's most significant difficulties during the past several decades. Moreover, it continues to be a major cause of death on a global scale. Worldwide, 19.3 million cancer diagnoses are anticipated in 2020, with about 10 million associated deaths (Rehman and Saba 2014). There are over 150 distinct types of cancer, and currently no practical solutions for treating them in their early stages (Alyami et al. 2022b; Ragab and Alyami 2023). Therefore, early cancer identification is the most promising method of increasing a patient's survival likelihood. With improvements in the medical profession and its technology, CAD systems have shown remarkable performance. CAD is a detection system that employs machine learning techniques and image processing using computer vision (Javed and Rahim 2020).

**Fig. 2:**
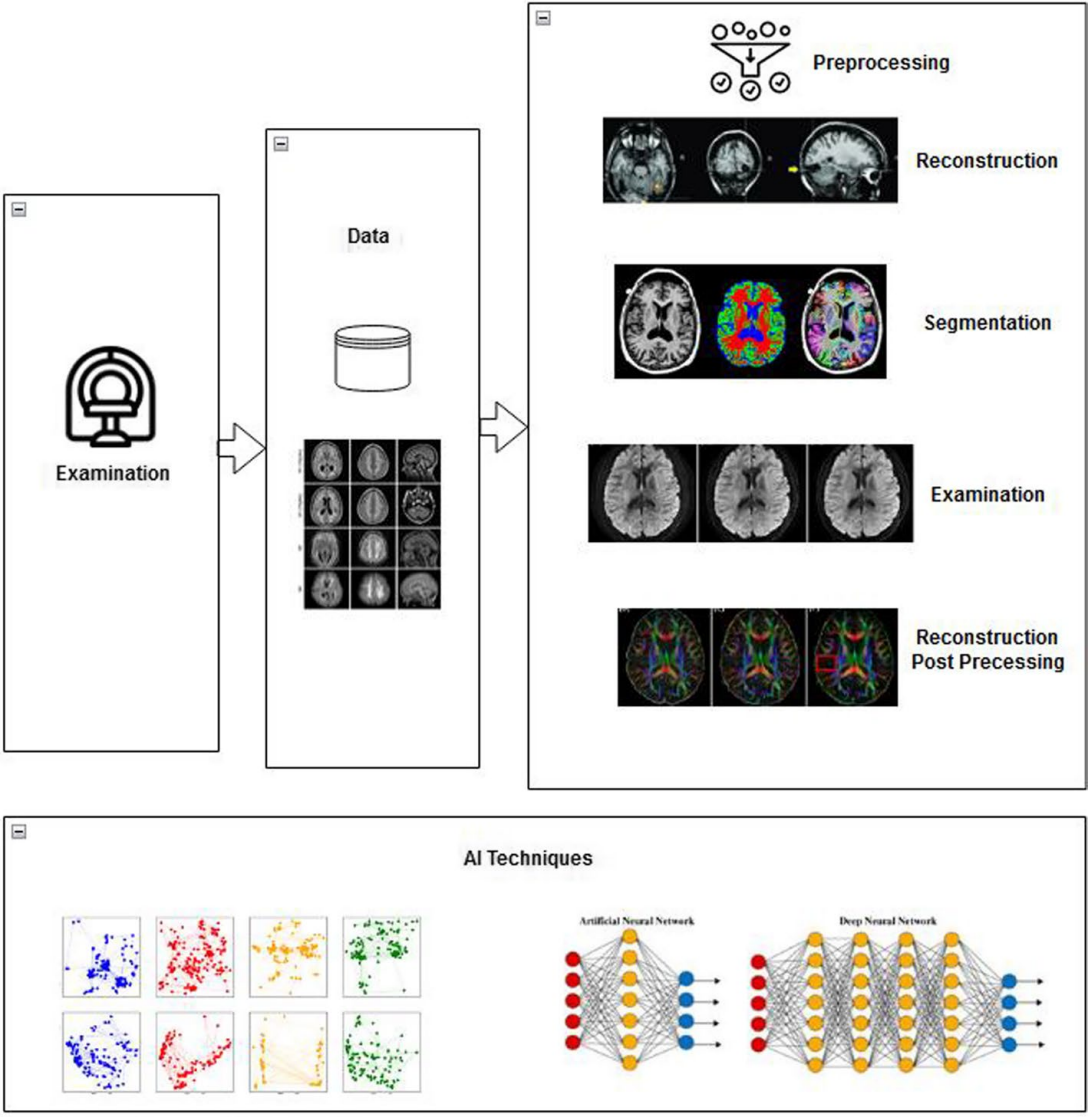
Overview of AI-based techniques and big data applications in medical imaging

Early detection of lung abnormalities is also critical for risk reduction and effective treatment. Unfortunately, these malignancies cause around 85% of lung cancer cases. Therefore, numerous researchers have proposed various methodologies to diagnose cancer from (Magnetic Resonance Imaging (MRI) scans. However, most reported works focus on four basic steps for cancer detection: image preprocessing, segmentation, feature selection from segmented images, and classification into cancer or non-cancer images (Muhsin et al. 2014; El Nawar et al. 2019). Figure 2 exhibits an overview of AI-based approaches with big data applications in medical imaging analysis and cancer diagnosis.

Breast cancer is common cancer among women worldwide. Since they offer significant phenotypic information, histopathological images are critical in diagnosing and treating breast tumours (Husham et al. 2016). Visual evaluation of histopathological samples is the standard for identifying breast cancer, but it's a hard approach. A precise breast cancer diagnosis is important for timely treatment (Iftikhar et al. 2017a). Efficient identification reduces pathologist effort and diagnosis ambiguity. Mammography, MRI, FNAC and histopathology are the most frequently used cancer screening and diagnosis procedures. The histopathology approach involves removing contaminated breast tissue and examine it under a microscope (Meethongjan et al. 2013; Saba and Rehman 2013). Deep learning models have proven successful in clinical diagnosis, medication discovery, frequency modelling, and optimization. The similarity of breast cancer histopathologic images and infected tissues make cancer identifying process crucial. A CAD system rapidly analysis images and delivers a diagnostic opinion, improving pathological decision-making accuracy (Marie-Sainte et al. 2019; Alnazer et al. 2021).

**Fig. 3:**
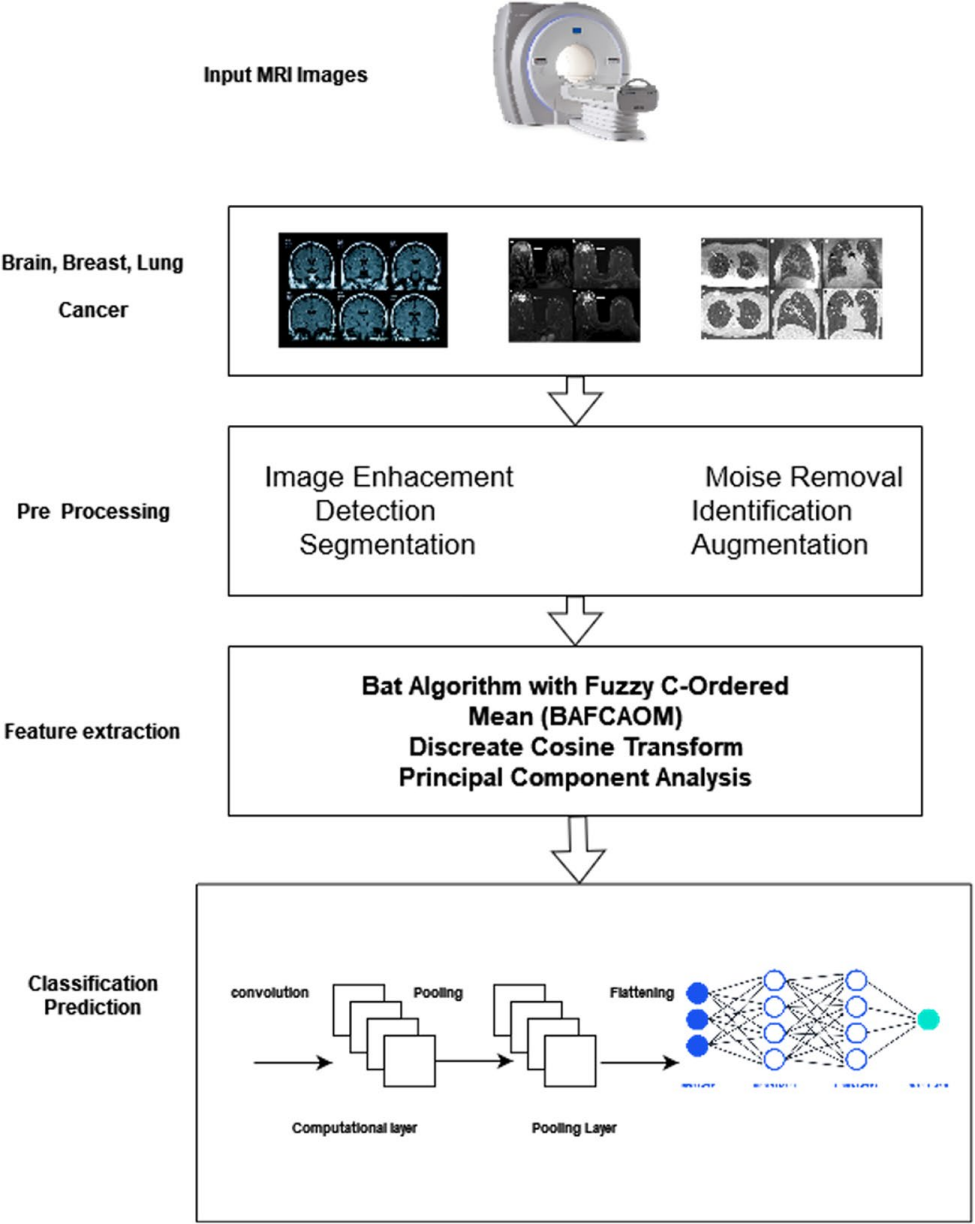
Cancer classification process using ML & DL

Bhandary (El Nawar et al. 2019) proposed two distinct deep learning approaches to examine lung pneumonia. To categorize chest CT scans into normal and pneumonia classes, first Modified AlexNet (MAN) is implemented. The categorization is carried out in the MAN using the SVM, the results with Softmax classifier. Additionally, SVM performance was compared to various pre-trained deep learning systems, including AlexNet, VGG16, VGG19, and ResNet50. The second deep learning effort integrated handcraft and deep features into the MAN to increase lung cancer classification accuracy and performance evaluated on LIDC-IDRI benchmark dataset. Classification accuracy (97.27%), sensitivity (98.09%), specificity (95.63%), precision (97.80%), F1 score (97.95%), and AUC (0.995%) are all achieved. According to the confusion matrix, the classification rate for normal and pneumonia features was 96.3% and 3.7%, respectively. Iftikhar et al. (2017a) recently researched pre-trained xception and deeplabv3+ construct semantic models. The model was trained on input images using ground masks to better segment ultrasound breast images into benign/malignant classes. The segmentation model's accuracy was more than 99%. The segmented and histological breast images are transmitted to a 4-qubit quantum circuit with a six-layered design to identify breast cancer. Yurttakal et al. (2020) constructed a model based on multi-layer CNNs and conducted trials on improved MRI. The dataset comprises 200 MRI scans of breast cancer obtained from a hospital in Istanbul, Turkey. They achieved 98.33% accuracy, 100% sensitivity, 96.9% specificity, 96.55% precision and 0.0167% loss. However, 3.4% of benign images were incorrectly categorized as malignant. Sharif et al. (2019) technique for multistage mitotic cell identification based on faster region convolutional neural networks (Faster R-CNNs) and deep CNNs. They employed two publicly available datasets (ICPR 2012 (MITOS-ATYPIA-12) and ICPR 2014 (MITOS-ATYPIA-14)) of breast cancer images. The experimental findings indicate that the technique achieved 87.6% precision, 84.1% recall and 85.8% F1-measure on ICPR 2012 dataset and 84.8% precision, 58.3% recall and 69.1% F1-measure for the ICPR 2014 dataset. Figure 3 presents the cancer classification process using machine and deep learning models.

Dermoscopy is the usual initial detection technique mainly employed to examine melanocytic lesions. However, as infrared thermal imaging is a non-invasive imaging technology that maps the temperature of the skin's surface related to pathological states, such as tumourous lesions, it may be a useful tool for all types of skin cancer (Escorcia-Gutierrez et al. 2022). Table 1 presents state-of-the-art CAD systems for cancer diagnosis and compares their performance on benchmark datasets.

**Table 1:** Performance comparisons of cancer classification using different classifiers

Refs.	Cancer pathology	Methodology	Features	Database	Performance
El Nawar et al. (2019)	Lung cancer	AlexNet (MAN) MAN	Support Vector Machines Principal Component Analysis	X-ray images LIDC-IDRI	Accuracy (97.27%), sensitivity (98.09%) specificity (95.63%), precision (97.80%), F1 score (97.95%) AUC (0.995%)
Husham et al. (2016)		Wavelet Recurrent Neural Network	Not mentioned	Lung images	sensitivity 93.75%, specificity 66.67%, and accuracy 84% for training data and sensitivity 88.24%, specificity 75%, and accuracy 84% for testing data
Iftikhar et al. (2017a)	Breast cancer	4-qubit-quantum circuit with six-layered architecture	Deep features	histopathological dataset	99%
Yurttakal et al. (2020)		Multi-layer CNN	Not mentioned	MRI	98.33% accuracy, 100% sensitivity, 96.9% specificity, 96.55% precision, and 0.0167 loss
Sharif et al. (2019)		Fusion (RCNN and CNN)	Not mentioned	ICPR 2012, ICPR 2014 [MITOS-ATYPIA-14] histopathology images	87.6% precision, 84.1% recall and 85.8% F1-measure on ICPR 2012 database, and 84.8% precision, 58.3 recall and 69.1% F1-on ICPR 2014 database
Escorcia-Gutierrez et al. (2022)	Skin cancer	Ensemble learning and deep learning	Thermal parameters from thermograms of lesions	Infrared thermal imaging	96.65% precision, 94.11% recall, 95.36% f1-score, 91.85% ROC (AUC)
Khan et al. (2019)		Neural network (NN) [DSL-1]	Not mentioned	Basal Cell Carcinoma (BCC)	62% sensitivity and 83% specificity
Andreeva et al. (2021)	Brain tumour	Support vector machine	Discrete cosine transform-based	MRI and SPECT images	96.8% accuracy, 95% precision, 94% recall, 93% specificity, 91% F1 score
Diab et al. (2020)		Bat Algorithm with Fuzzy C-Ordered Means (BAFCOM) Enhanced Capsule Networks (ECN)	Region of Interest (RoI)	MRI	95.81% accuracy, 94.83% precision, 94.34% recall and 94.85% F1-score
Al-Koussa et al. (2020)		SVM, KNN, DT classifiers fusion	Adaptive histogram equalization	MRI & SPECT	Accuracy 96.8%, precision 95%, recall 94%, specificity 93%, F1 91%
Saba et al. (2012)		3D CNN	Deep features extraction	BRATS MRI	98.32% highest accuracy

**Fig. 4:**
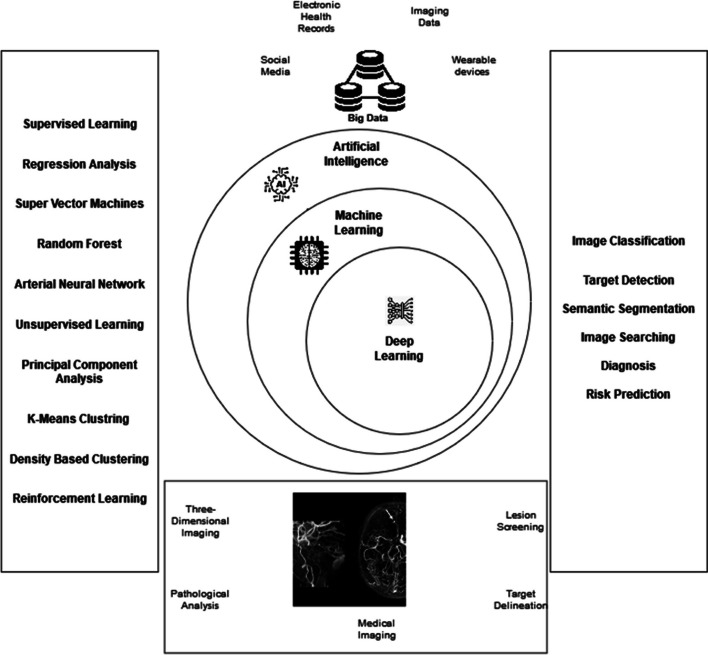
Different AI techniques for medical imaging analysis & diagnosis

MR images are assessed for malignancies in clinical practice and monitor therapy effectiveness. Thus, segmenting brain tumours effectively using MRI data is critical for clinical diagnosis and therapy planning. Preprocessing significantly improves images' quality and improves classification accuracy. Due to brain tumours' varying shape, size and texture, segmenting the Magnetic Resonance Imaging (MRI) is very complicated during their examination. However, physicians and radiologists can quickly identify and classify cancers if a system integrates CAD. Al-Koussa et al. (2020), used the contrast-restricted adaptive histogram equalization approach to preprocess input images such as MRI and SPECT. In the next stage, discrete cosine transform-based fusion is applied to classify images into benign and malignant brain tumours. They also analyzed the performance of AI methods such as SVM, KNN, Random Forest (RF) and decision tree classifiers employed for feature extraction from enhanced medical images. The highest accuracy of the SVM classifier was 96.8%, 95% precision, 94% recall, 93% specificity and 91% F1 score reported. Saba et al. (2012) proposed an automated segmentation of tumours from MRI and improved the effectiveness of segmentation and classification. In their work, MRI was evaluated using the Enhanced Capsule Networks (ECN) approach to determine stage of brain tumour. Finally, the ECN algorithm evaluated the proposed approach's performance by discriminating between two kinds of tumours in MRI, in addition to the recommended ECN classifier's accuracy (95.81%), precision (94.83%), recall (94.34%), and F1-score (94.585%). Figure 4 presents an overview of AI techniques in cardiovascular imaging analysis and cancer classification.

XmasNet, an innovative deep learning framework, was created for the purpose of classifying prostate cancer lesions. This architecture utilizes convolutional neural networks and leverages the 3D multiparametric MRI data provided by the PROSTATEx competition. The XmasNet model was trained completely, using techniques like 3D rotation, and slicing to add more data and better capture the 3D features of the lesion. XmasNet demonstrated superior performance compared to conventional machine learning models that relied on artificially generated features for both the training and testing datasets (Liu et al. 2017). XmasNet demonstrated superior performance compared to 69 methodologies from 33 rival teams in the PROSTATEx challenge, achieving the second-highest area under the curve (AUC) value of 0.84 during testing. This study demonstrates the immense potential of deep learning in the domain of cancer imaging. It's very common to get prostate cancer, which is the third most common type of cancer-related death in North America. A lot of studies have been done on diffusion-weighted magnetic resonance imaging (DWI) as a precise way to find prostate cancer when it is used with computer-aided detection (CAD) systems. CNN is very good at computer vision tasks like finding objects and separating them into groups. So, researchers in medical imaging are looking into different CNN designs as possible ways to make CAD methods for finding cancer more accurate. As part of our study, we created and ran a computerized CNN system that looked at the axial diffusion-weighted imaging images of each subject. The goal was to find prostate cancer (PCa) cases that are important for medical reasons. The dataset was made up of DWI scans of 427 patients, including 175 people who were identified with PCa and 252 people who did not have PCa. Out of the 427 patients that were used in the training phase, 108 were set aside and not used to test how well the planned pipeline would work. There was an area under the receiver operating characteristic curve (AUC) of 0.87 (95% CI 0.84–0.90) at the slice level and 0.84 (95% CI 0.76–0.91) at the patient level for the suggested pipeline (Yoo et al. 2019).

Prostate cancer is highly prevalent and lethal among men in the United States. The presence of intricate masses poses challenges for radiologists in detecting prostate cancer. Several contemporary prostate cancer screening technologies were developed, but they proved to be useless. This study utilizes transfer learning to construct a resilient deep-learning CNN. Comparisons are conducted using decision trees, SVM kernels, and Bayes. They get morphological, entropy-based, texture, SIFT (Scale Invariant Feature Transform), and elliptic Fourier descriptors from cancer MRI databases so that GoogleNet and machine learning classifiers can learn how to use them. Performance is evaluated by calculating specificity, sensitivity, positive and negative predictive values, a false-positive rate, and the receiving operating curve. The CNN model, specifically GoogleNet, achieved the highest performance when utilizing transfer learning. We achieved satisfactory outcomes using decision trees, support vector machines with RBF kernels, and Bayes. However, deep learning produced exceptional results (Abbasi et al. 2020).

### Neurological imaging

"Neurological disorders" refers to illnesses affecting central nervous systems. Muscle weakness, paralysis, convulsions, discomfort, poor coordination and loss of consciousness are all typical symptoms. Less than 600 neurological illnesses, including brain tumours (Nazir, et al. 2019). Artificial intelligence (AI) in medical imaging is a promising technique. MRI can offer information about the brain's anatomical structure and detailed multi-parameter information on its function and metabolism. Due to its excellent spatial resolution, structural magnetic resonance imaging (sMRI) and functional magnetic resonance imaging (fMRI) have achieved significant advances for investigating brain organization. Technological advancements such as medical image analysis using deep learning may all be applied to diagnose various neurovascular or other neurological conditions. These approaches include increased spatial resolution, decreased acquisition time, the ability to characterize discoveries using standard MRI (Saba et al. 2018b).

Epilepsy is often diagnosed by a neurologist but may be challenging early. Paraclinical data derived from magnetic resonance imaging and electroencephalography may assist physicians in making an epilepsy diagnosis and initiating therapy early. However, electroencephalogram acquisition and interpretation are time-consuming and may be costly since they need the services of skilled professionals. Automated identification of seizure activity correlations may be a solution. This article offers a supervised machine-learning strategy for classifying seizure and nonleisure recordings using a 342-record open dataset. Their findings demonstrate an improvement of up to 10% in most situations over previous research (Amin et al. 2020a).

**Fig. 5:**
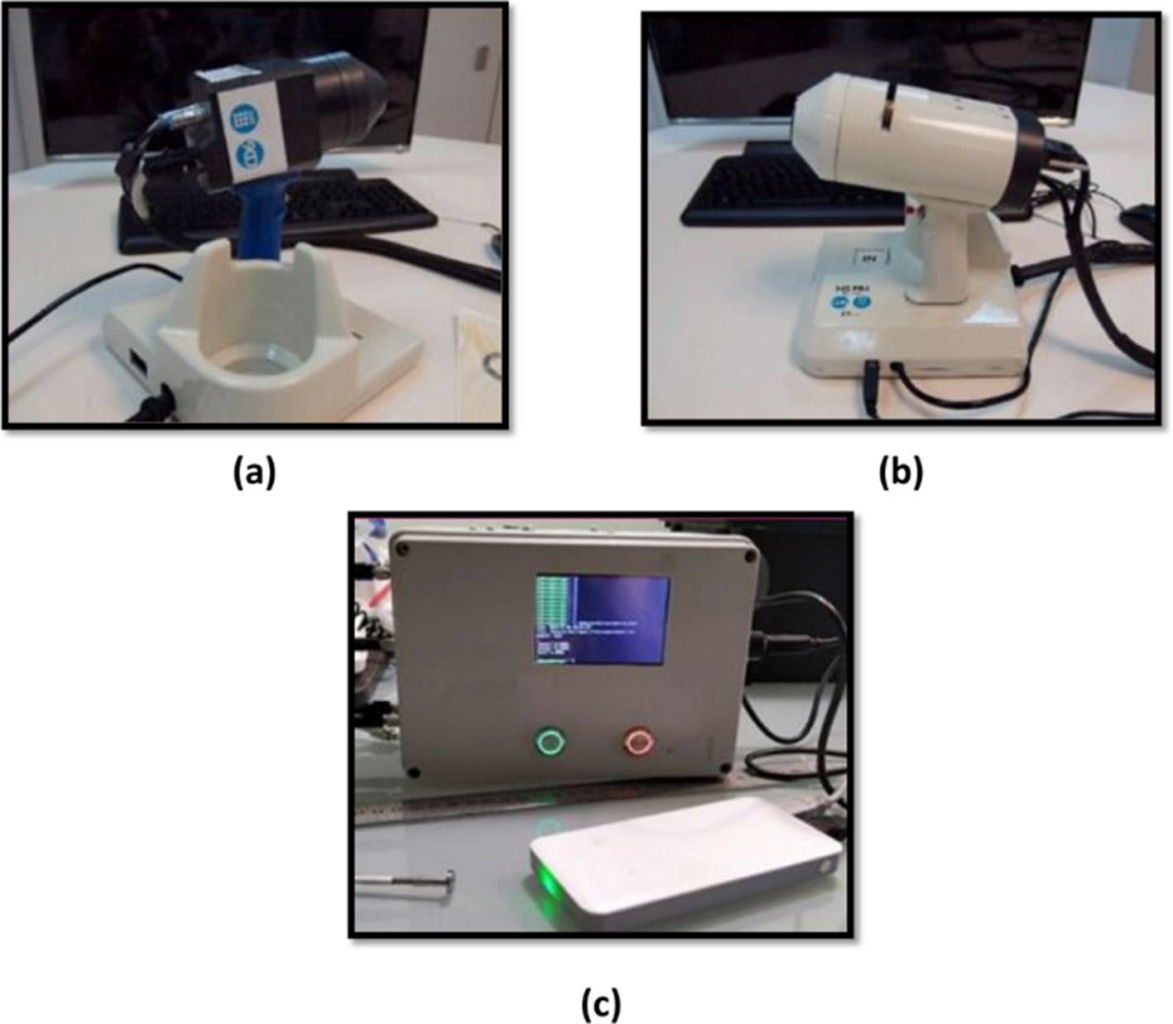
Diagnostic devices for skin cancer **a** exNIR multispectral MS device **b** VIS–NIR MS device **c** fluorescence spectra recording with DSL-1

Ojeda et al. (2019) convolutional neural network (CNN) was validated using 7112 non-contrast CTs gathered from two metropolitan hospitals and 99% specificity, 95% sensitivity, and 98% accuracy were reported. SMRI is commonly utilized in clinics to identify neurological and psychiatric illnesses and brain diseases. Bahmad et al. (2020) sought to determine if anatomical patterns in the brains of people with autism spectrum disorder (ASD) may be biomarkers to diagnose and assess ASD in clinics. A unique histogram-based morphometry (HBM) classification structure was created. Features were extracted from sMRI data using 3D histogram of oriented gradients (HOG). The proposed HBM structure produced more than 75% AUC on the benchmark dataset. Bar-Sela et al. (2019) used hyperspectral imaging (HSI) as optical imaging to extract additional information from scanned tissue. Since human observers cannot visually analyze HSI images, they studied the potential of HSI in twelve in vivo specimens using ANN and CNN. The proposed system utilizes a hybrid 3D–2D CNN technique to jointly extract spectral and spatial information from in vivo HS brain cancer hyperspectral images. In addition, proposed network using 2D CNN and 1D DNN with traditional classification techniques attained 80% accuracy. Figure 5 exhibits different diagnostic devices for skin cancer.

**Fig. 6:**
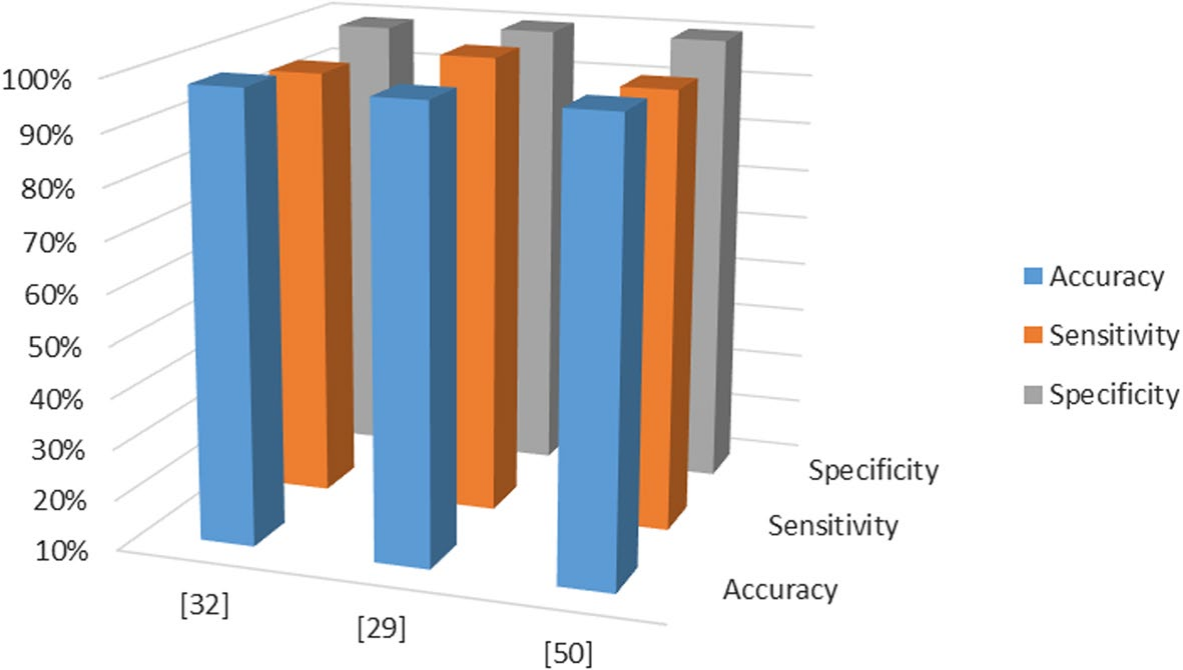
Performance analysis of different AI-based methods in neurological imaging

Normal Pressure Hydrocephalus (NPH), or Atypical Parkinsonian syndrome, is the most prevalent neurological condition among the elderly (Amin et al. 2020b; Ragab et al. 2022a). This syndrome manifests Parkinson's disease (PD) signs such as short memory, dementia, bladder control issues etc. MRI is the most appropriate technique for detecting aberrant cerebrospinal fluid accumulation in the brain's cavities or ventricular, the primary cause of NPH (Amin et al. 2019). Siegersma et al. (2019) developed an automated deep learning-based system to identify hydrocephalus effectively utilizing biomarkers for NPH segmentation and classification (NPH-SC). To increase accuracy, image preprocessing is performed. Alzheimer's disease is one of crucial prevalent type of dementia. It is incurable neurological disease causes gradual mental decline. On 3D brain MRI, an automated Alzheimer's disease (AD) detection convolutional neural network (CNN) system based on deep learning was developed. Each of the three groups in the structure comprises three convolutional, pooling, and normalization layers. MR images from the DNI dataset were employed for training and testing purposes. The data included MRI scans of 47 Alzheimer's patients and 34 healthy controls. The experiment claimed 97.65% AD identification accuracy, 100% sensitivity, and 93% specificity. Table 2 presents a performance analysis of different machine learning approaches for neurological disorders detection on benchmark datasets and their graphical comparisons in Fig. 6.

**Table 2:** Performance Analysis of Neurological Disorders

Refs.	Methodology	Features	Database	Advantages	Limitations
Nazir, et al. (2019)	Convolutional neural network (CNN)	Automatic deep features	CT images	Specificity 99%, sensitivity 95%, and accuracy 98% claimed	No benchmark datasets were experimented. Only local dataset used. Difficult to generalize results
Amin et al. (2020a)	SVM and 3D–2D hybrid CNN combination	Spectral and Spatial features	vivo HS brain cancer dataset	80% accuracy attained	No benchmark dataset was experimented. Also accuracy is low
Saba et al. (2018b)	2-level histogram-based morphometry (HBM)	3D histogram of oriented gradients (HOG)	sMRI	(AUC) of > 0.75 in each dataset, highest AUC of 0.849 in case of ETH site	Traditional machine learning used that is not suitable for large datasets. Also accuracy is low
Ma et al. (2022)	Normal Pressure Hydrocephalus segmentation and classification (NPH-SC) model and CCN	Skull features and segmentation by marker-based watershed approach	MRI Brain	NPH-SC model: sensitivity 96%, specificity 100%, validation accuracy 97%. CNN test accuracy 98%	Experiments are performed on small dataset & validity of the approach seems not good on large dataset
Mathur et al. (2020)	CNN	geometric data augmentation techniques	MRIs	Accuracy 98.8% and specificity 99%	No benchmark dataset used & experiments performed on augmented data
Jabeen et al. (2018)	Feedforward multilayer neural network	PCA + ANN	MRIs	Accuracy 75%	Small dataset employed for experiments

### Cardiovascular imaging

The number of deaths caused by cardiovascular disease (CVD) in US was 17.6 million in 2016 which was 14.5% higher from 2006 (Rahman et al. 2023; Ejaz et al. 2021). CVD has a major economic cost for society, estimated at $351.2 billion in the United States, and has long-term impacts on patients' quality of life. The EU estimates that the total annual cost will be €210 billion. The inflammatory disease of the blood vessels, known as atherosclerosis, is the leading cause of CVD. Endothelium dysfunction causes atherosclerosis by causing damage to the blood vessel's inner surface's thin wall, creating increasingly complex lesions and fatty streaks inside artery walls (Iftikhar et al. 2017b; Ragab et al. 2022b).

Ece et al. (2022) research included 600 patients, 300 females and 300 males, diagnosed with IHD due to data acquired from reports of patients who had Coronary angiography CAG at the Frat University Hospital. The categorization yielded accuracy, precision, sensitivity, specificity and F1-score performance scores. KNN algorithm had the best success rate of all the tested algorithms. In the second success rate, SVM came in second. KNN had an RCA success rate of 83%, 95% accuracy, 82.2% sensitivity, 85.4% specificity and 87.8% f1 score. In the instance of KNN, the success rate of LCx 76%, accuracy 70.6%, sensitivity 80.2%, specificity 73.7% and f1 score 75.1%. In KNN, the success rate of LAD 73%, sensitivity 72.7%, accuracy 75%, specificity 74.5%, and the f1 score 73.6% reported.

Automatic segmentation of myocardial fibrosis or scar might significantly improve predicting and treating malignant ventricular arrhythmias in individuals with cardiovascular disease. Zabihollahy et al., (2018) employed CNN based approach and claimed 94.50% segmentation accuracy. However, their findings for scar segmentation are more consistent with human expert segmentation than various intensity threshold-based methods. The suggested method's primary drawback to identify endocardial and epicardial boundaries manually. Table 3 briefly describes state-of-the-art AI-based approaches for cancer detection.

**Table 3:** State of the art AI—based models for cancer diagnosis by radiology images analysis

Refs.	Approach	Modality	Brief Description
Ejaz et al. (2021)	Unsupervised learning	Echo	Identified diastolic dysfunction patients' left atrial and ventricular strain clusters
Iftikhar et al. (2017b)	Supervised learning	Echocardiography Imaging	Complications were predicted using clinical and echocardiographic factors
Ragab et al. (2022b)	Support Vector Machine (SVM)	SPECT	Using a support vector machine technique, improved SPECT to identify coronary artery disease
Ece et al. (2022)	Deep learning	CT	Analyzed the performance of automated and manual assessments of the architecture and functions of the left and right sides of the heart
Zabihollahy et al. (2018)	Machine learning	MRI	RVR reconstruction using echocardiography and cardiac MRI was more accurate than straight cardiac MRI
Bustin et al. (2020)	Deep learning	MRI	Evaluated ventricular contours with hand tracing
Dhasarathan et al. (2023)	Deep learning	X-ray	Simple CapsNet

### Abdominal imaging

MRI plays a vital role in abdominal imaging. An abdominal MRI provides detailed pictures of the belly area from many views to describe tissue damages and finding abnormalities in patients. MRI has many advantages including the absence of ionizing radiation, improved soft tissue contrast, speed, high contrast resolution, and multi-planar capability (Alyami et al. 2015). Breath-holding, patient sedation, respiratory gating and image post-processing are all common ways to decrease or remove human abdominal issues. To begin with, a minimal sample size was chosen.

Moreover, only MR images were employed as the classification inputs (Mughal and Muhammad 2018). The most often utilized imaging methods in abdominal pathology are CT scans and MRI. Due to potential nephrogenic systemic fibrosis, contrast delivery in MRI is limited in individuals with renal insufficiency. MRI contrast media allergies are uncommon and minor compared to CT contrast media allergies (Jabeen et al. 2018). Unfortunately, imaging procedures such as CT or MRI may have serious side effects for patients or have contraindications that restrict their diagnostic potential. Tissue heating generated voltages in the neurostimulator and lead dislodgement may all occur due to MRI exposure. Under the impact of MRI, metallic foreign bodies might shift, causing harm to critical tissues such as nerves, arteries and the eye. MRI is impossible if the brain aneurysm clips are constructed of ferromagnetic materials. The examination is feasible if the clips are titanium alloy (Kurdi et al. 2023; Naz et al. 2023).

Anderson et al. (2021) suggested an automated blood loss prediction approach based on MRI uterine image to estimate the quantity of bleeding in the caesarean procedure of patients with Pernicious Placenta Previa (PPP). The DeepLab-V3+ network was first utilized to segment the original MRI abdomen image to produce the uterine area image. The Visual Geometry Group Network-16 (VGGNet-16) network was then used to train the uterine area image and the related blood loss data. The blood loss level categorization model was discovered. The suggested technique attained 75.61% accuracy, 73.75% sensitivity and 77.46% specificity from the dataset of 82 positive samples and 128 negative samples.

Emergency rooms commonly seek abdominal imaging to diagnose the source of a patient's abdominal discomfort. Both an AXR and an ACT are frequently ordered for these patients in three Nova Scotia hospitals. Research suggests that an abdominal computed tomography (ACT) exhibits superior diagnostic quality than an abdominal x-ray series (AXR) and is eventually prescribed. Imaging modalities, such as LDCT and ASIR, may lower the radiation exposure of an ACT while retaining diagnostic quality. The outcomes of this study highlight the need for additional instruction on correct test ordering practice for AXR versus ACT (Saba et al. 2022; Hassan et al. 2021).

Modern imaging methods may quickly reveal tumour thrombus inside the abdominal veins. Abdominal vein tumours could be primary or secondary to endoluminal extension from other organs. Primary venous tumours are benign or malignant ones arising from the vascular endothelium or smooth muscle cells inside the artery wall. Although leiomyosarcoma is an uncommon tumour, it is considered one of the most prevalent primary tumours of the abdominal veins (Rehman et al. 2021a). Compared to their initial counterparts, secondary tumour extension into the lumens of the abdominal veins seems more prevalent. The proclivity for venous invasion is well documented in tumours from the liver and kidneys; nevertheless, various other malignancies may also extend into the venous lumen (Rehman 2020, 2021).

This study employed deep learning approaches to automatically combine PET and CT characteristics for 3D whole-body MM bone lesion identification. In order to partition and classify the abnormalities, two convolutional neural networks (CNNs), namely V-Net and W-Net, were employed. We used advanced PET simulation techniques to make digital phantoms that were used to test how well deep learning could find lesions on 68 Ga-Pentixa for PET/CT. The proposed methodologies Abdominal segmentation was trained on a convolutional neural network with the U-Net architecture in this retrospective study (Weston et al. 2019).

The network was tested on an additional 270 CT exams after it was trained on a dataset of 2430 two-dimensional CT exams. Furthermore, the assessment was carried out on a separate dataset comprising 2369 individuals with a diagnosis of hepatocellular carcinoma (HCC). CT scans were performed between 1997 and 2015. The students were 67 years old on average. Male patients ranged in age from 29 to 94 years old, with an average age of 67 years. Female patients ranged in age from 31 to 97 years old, with an average age of 66. A two-way analysis of variance was used to assess the segmentation performance, and Bonferroni correction was included to identify any discrepancies (Liu et al. 2018). These discrepancies were further evaluated utilizing real 68 Ga-Pentixa for PET/CT images of patients with MM. The study showcases the effectiveness of a deep learning-based method that surpasses the existing clinical techniques used for MR imaging-based attenuation correction (MRAC). Specifically, deep MRAC led to a PET reconstruction inaccuracy of less than 1% in the majority of brain regions (Akagi et al. 2019).

## Limitations, pitfalls and future challenges

AI applications generally mimic human intelligence to solve issues or make judgments. With its precision, cost-effectiveness, and dependability, AI offers several benefits. However, AI still has significant limits, particularly regarding medical applications (Doumit et al. 2022; Abunadi et al. 2022a). There are now initiatives ongoing to enhance imaging mode comparability. The first problem to be 'solved' is expected to be automated segmentation or extraction of imaging characteristics, which will assist in standardizing and speed up the analysis of big datasets. Another area for improvement is that the datasets mentioned in the research are often tiny. Furthermore, investigations must involve a broad spectrum of patients (Elias-Rizk et al. 2020; Sajjad et al. 2021).

AI algorithms developed and evaluated at one institution may not apply to other cohorts. As a result, all models must be externally validated across different centers and imaging providers before broad clinical implementation. Furthermore, AI models may benefit from continual updating utilizing huge and dynamic data sets as patient features, disease patterns, and image capture and reconstruction methodologies change over time (Mujahid et al. 2022). In 2019, for example, research examined the utilisation of three CNN algorithms for segmenting forty-four patients' MRI data with brain tumour across two universities. The model's performance was significantly worse when applied to data from many universities than from a single university in this investigation. This was obvious in segmenting the whole tumour and its constituent parts. In addition, the study indicated that using data from several universities for training CNN models had a significant impact; however, the reasons for this remain unknown (Saba et al. 2018; Hussain et al. 2020).

In the event of varied imaging resolutions, pulse sequences, acquisition trajectories, magnetic field intensity, MR vendors, or clinical locations, these reconstruction approaches' generalization potential and efficacy should be further examined. For example, while various neural networks for different tests might be pre-trained, a DL model's poor generalization ability to diverse sequence conditions, anatomy, physiology, or specific diseases would restrict its application into clinical practice. Furthermore, the heart's respiratory solid and cardiac motion during MR capture may severely degrade image quality, causing blurring and ghosting-like aberrations (Fahad et al. 2018; Abunadi et al. 2022b). As a result, most machine learning relies on previous medical data obtained using training datasets, leading to overfitting and selection bias. Furthermore, models created on training sets need to generalize better to anonymous data, known as overfitting. Using machine learning methods for echocardiography, CTCA, SPECT and CMR is still a new research domain (Saba et al. 2018b; Fahad et al. 2018). With machine learning algorithms, there is a risk of false discovery. This is more likely to happen with smaller data sets. Large, well-structured, labelled datasets are required to train and validate novel approaches. In addition to data availability, data quality is critical for developing accurate AI systems. A more extensive, more diverse data set would be necessary to generalise the approach. Finally, to achieve optimum models, the impacts of ambient noise on unfiltered signals and hence the derived predictors must be investigated further (Shahzad et al. 2021; Rehman et al. 2021b). Because there aren't enough medical images with plenty of labels in real life, and labelling such data can only be done by physicians, studying the localization of lesions under poor supervision is useful. Based on CAM theory, the single classification network completes finding cardiovascular lesions and aids the model's interpretability. However, the lower accuracy emphasises further research in this domain (Haimed et al. 2021; Chalah et al. 2019).

Using machine learning in health care, inappropriate dichotomisation and calibration are well-known issues. Privacy, security, and data breaches are all concerns that algorithm development faces. Furthermore, current frameworks or norms must define how to design algorithms using sensitive medical data, cooperate across institutions, or own algorithms developed from patient data. European privacy rules have become stricter with the European Union's implementation of the General Data Protection Regulation (GDPR) in 2016. The General Data Protection Policy (GDPR) is a European Union regulation on data protection and privacy that applies to all persons in the European Union and the European Economic Area. It covers topics such as individual permission to use personal data, what to do during a data breach, fines for failing to follow the appropriate process, and so on. The growing use of AI in health care, particularly with patients' electronic medical records, includes social demographic data such as social security numbers and medical coverage information alongside medical data. Better data protection legislation is required in the USA. Regrettably, there are currently no universal regulations or norms in place. Other barriers to broad usage include a lack of standardization, applicability, repeatability and legal duties.

ML algorithms' "black box" aspect has always been their Achilles heel and hindered their adoption. However, these programmes need to be built with ethics in mind. Physicians must be adequately trained in these areas of machine learning algorithms to improve the medical industry. Medical students should be exposed to ML early in their medical school careers.

## Open access benchmark datasets

This section describes several benchmark datasets used in abdominal imaging, cardiovascular, neurological and multi-organ cancer detection.

- Wisconsin Dataset for Diagnostic Breast Cancer (WDBC) comprises 569 cases, 212 malignant and 357 mild cases. It was developed at UC Irvine.
- MIAS dataset composed of 322 mammograms, 189 regular and 133 abnormal. "Architectural distortion, asymmetry, calcification, spiculated masses, circumscribed masses, and different images."
- Breast Histology Bioimaging Challenge Dataset: Composed of 249 annotated H & E images of normal, benign, in situ carcinoma, and aggressive cancer, the collection contains an upgraded test file with 16 images at 2040 1536 pixels resolution and 200 × magnification (Elias-Rizk et al. 2020).
- OASIS-3, OASIS-2, and OASIS-1 are composed of 373, 434, and 2168 MR images of 150 participants/patients.
- MIRIAD is composed of MRI of Alzheimer's patients and healthy older persons. With mild-moderate Alzheimer's disease, this database has 46 patients and 23 healthy controls. These data come from 55 people with Parkinson's and 23 people with PD-related disorders who participated in the NTUA Parkinson study. There are around 42,000 images available for academic use.
- From Neuromorphometrics, Inc., Scotts Valley, California, USA, MICCAI-2012 contains 35 T1-w MRI volumes with manual segmentation of 134 structures. It is usually utilised to segment tissues and structures. This collection started in 2012 with 80 real and fraudulent instances. Recently, the amount of training and testing data has increased.
- PH2 Skin Cancer Dataset comprises two hundred dermoscopic images, forty melanoma and one hundred sixty nevi.
- MED-NODE Skin Cancer Dataset contains 170 clinical images, 70 melanoma and 100 nevi.
- Asan Skin Cancer Dataset contains 17,125 clinical images of 12 Asian skin disorders.

## Conclusion

Finally, AI's use in medical imaging offers new possibilities in the early detection and diagnosis of numerous cancers and improved clinical choices, outcome prediction and prognosis assessment. However, finding the right mix between completely autonomous AI and medical oversight is a novel and challenging task. The issues of complexity and expense in optimum diagnosis and monitoring therapy exist and one viable answer is to improve hardware and software. The problem with vast and varied datasets is the assimilation of experts and AI-based solutions and the need for more open-source datasets. Developing AI algorithms as open-source and multicenter partnerships is a feasible approach for integration.

Furthermore, treatment is more consistent since it is based on an automated process. With AI performing jobs more quickly, clinicians have more time to teach patients and build deeper physician relationships instead of tedious activities like data entry and electronic health records. Although AI significantly assists in decision-making, doctors still make the ultimate clinical decision. To develop highly competent solutions in medical imaging including MRI and other radiology modalities  for cancer diagnosis at an earlier stage, particular requirements and quality control systems will be required in the future. In addition, concerns about the datasets employed and their size, variety of images from different races and ages must be addressed. The primary way AI may benefit imaging experts and physicians is via intelligent precision medicine technologies. AI might be the technology we have been waiting for to make imaging the keystone of healthcare, guaranteeing that all information included in medical images is properly utilized to enhance illness diagnosis, prediction and therapy. Unfortunately, we have yet to be there to achieve this aim, AI might need to meet the highest standards and, perhaps more importantly, need the trust of both patients and experts.

## Data Availability

Not applicable.
